# Processes and Predictors in the Transition From Pediatric to Adult Epilepsy Care: A Retrospective Study at a Single Institution

**DOI:** 10.1016/j.pediatrneurol.2025.07.015

**Published:** 2025-07-28

**Authors:** Jasmine Sondhi, Esraa Ali, Krishna Trivedi, Stephanie DeCarvalho, Traci M. Kazmerski, Laura Kirkpatrick

**Affiliations:** a UPMC Children’s Hospital of Pittsburgh, Pittsburgh, Pennsylvania; b University of Pittsburgh School of Medicine, Pittsburgh, Pennsylvania

**Keywords:** Pediatric epilepsy, Epilepsy, Health care transition, Transition of care

## Abstract

**Objective:**

To characterize transition of care processes for patients with epilepsy in a single institution without a formal epilepsy transition of care program.

**Methods:**

We reviewed medical records for individuals with epilepsy who were at least 15 years old seen outpatient by pediatric neurology in 2019. We followed their records through 2022. We evaluated whether they had documentation of transition discussion, if they transferred to adult care, and/or if they were retained in adult care. Excluding individuals who died or were discharged, we performed logistic regression for transition discussion, transfer, and retention, adjusting for sex, age, race/ethnicity, distance from institution, zip code median household income, rurality, drug-resistant epilepsy (DRE), intellectual disability (ID), and technology dependence.

**Results:**

We evaluated documentation for 274 individuals (50% female, median age 18 years, 84% white, 1.5% Hispanic, 24% DRE, 25% ID). Excluding 14 who died and 28 who were discharged, 57 of 226 (25%) had a documented transition discussion and 115 (51%) transitioned (median age 20 years). Of 95 eligible individuals, 77 (81%) were retained in adult care. Predictors of transition discussion included older age (aOR: 1.44, 95% confidence interval: 1.22, 1.70) and absence of ID (aOR: 4.17 [1.51, 11.11]). Predictors of transfer included older age (aOR: 1.19 [1.02, 1.39]), documented transition discussion (aOR: 3.25 [1.48, 7.13]), and DRE (aOR: 2.41 [1.10, 5.28]). We identified no significant predictors of retention.

**Conclusions:**

Discussion of transition may promote transfer to adult care among people with epilepsy. Formal transition programs may be needed to optimize care processes.

## Introduction

According to Got Transition, a national repository of best practices on the transition from pediatric to adult health care maintained by the National Alliance to Advance Adolescent Health, health care transition is “the process of moving from a child/family-centered model of healthcare to an adult/patient-centered model of healthcare, with or without transferring to a new clinician.”^1^ Got Transition outlines Six Core Elements of an ideal health care transition process, which are intended for primary care, although they are also applicable to specialty care.^1^ The Six Core Elements are echoed in an eight-element process delineated in a consensus statement on the neurologist’s role in health care transition affirmed by the Child Neurology Foundation, Child Neurology Society, American Academy of Neurology, and American Academy of Pediatrics.^1,2^ The principles from Got Transition and the neurology consensus statement require both practice/institution-level and clinician-level care coordination.^1,2^ Formal transition programs in neurology have emerged as a strategy to implement effective, guideline-concordant transition processes, although such programs may not be common or widespread.^3–9^

However, little is known in the neurology literature about how the transition process succeeds or fails in the absence of a coordinated institutional/practice-based transition program. In the Division of Child Neurology at our institution—a tertiary/quaternary care children’s hospital serving a large catchment area—no formal transition program existed for any subspecialty until 2023. The transfer process relied on *ad hoc* individual clinician behaviors. Providers in our institution have previously reported highly variable transition practices, as well as an overall low degree of satisfaction in the transition of care process.^10,11^

As a needs assessment preparatory to initiating a formal transition program in epilepsy, we conducted this study to determine institutional performance on key transition metrics through retrospective analysis of electronic health records (EHRs). In addition, we aimed to identify predictors of outcomes to understand if our processes differently impacted patient subpopulations. We hypothesized that fewer than 50% of patients would have documented transition discussions in pediatric neurology and fewer than 80% of patients would have documented transfer to adult health care, of which fewer than 80% would retain in adult neurology.

We focus on epilepsy in this study because most neurology transition programs in the literature are disease specific.^4–9^ Furthermore, epilepsy is a paradigmatic, common condition in child neurology, with a high burden of morbidity and mortality, requiring ongoing health care and patient self-management, making a successful transition process critical for this condition.^12–14^ Extensive literature has outlined challenges in the epilepsy transition and transfer process.^15–19^

Although we describe practices and outcomes in a single institution, we present this study both as a replicable methodology for how an institution can assess transition and transfer processes internally and as an illustrative example of processes and outcomes in transition and transfer in the absence of a formal, systematic, institutional/practice-based initiative.

## Methods

We obtained a list of individuals who were seen by outpatient Child Neurology at our institution in 2019 who were at least 15 years old at the time of appointment and who were diagnosed with epilepsy based on International Classification of Diseases - 10 codes (i.e., G40, R56). We confirmed a diagnosis of epilepsy per International League Against Epilepsy criteria through manual review of the EHR.^20^ For eligible patients with epilepsy, we reviewed their EHR from their appointment in 2019 through the end of 2022. We determined if there was evidence of documentation of a discussion about transition of care in clinical documentation of a pediatric neurology visit. We also assessed if there was a documented transition summary note in the EHR.

We determined if the patient had transferred to adult epilepsy care based on EHR documentation, including either adult EHR documentation verifying transfer or pediatric EHR documentation indicating imminent transfer to a specific adult practice (largely from documentation of telephone messages). Of note, there are two major health systems that provide adult neurology care in our region. Our children’s health system is the only pediatric neurology provider for the region and houses a Level 4 epilepsy center. Of the two major health systems providing adult neurology care in our region, both house Level 4 epilepsy centers. One health system is directly affiliated with the children’s hospital as part of the same health system, whereas the other adult health system is not directly affiliated with the children’s hospital. However, both adult neurological practices accept transfer referrals from the children’s hospital. We as investigators at the children’s hospital have access to EHR documentation from both adult neurological systems.

For those patients who transferred and had visit documentation available, we also determined if the patient retained in adult health care, defined as having at least one appointment per year with at least one year between the first adult appointment and study end. We also recorded if patients died before transfer to adult neurology. In addition, we determined if patients were lost to follow-up before transfer, defined as no EHR documentation (either pediatric or adult) for a two-year period following their last documented appointment.

We extracted patient demographics, including age in 2019, sex, race, ethnicity, documentation of a co-occurring diagnosis of intellectual disability (ID), documentation of use of technologies such as tracheostomy or feeding tube, presence of drug-resistant epilepsy (DRE) per International League Against Epilepsy criteria,^21^ use of the ketogenic diet, use of a vagus nerve stimulator, use of a responsive neurostimulator, and history of resective/ablative surgery for epilepsy treatment. We documented zip code, which we used to determine zip code distance to medical center, zip code median household income as a proxy of socioeconomic status, and zip code rurality based on Health Resources and Services Administration metrics.^22^

We used STATA (StataCorp, College Station, TX, USA) for all statistical analyses. We calculated descriptive statistics. We performed logistic regression analysis to identify significant predictors of key outcomes, including documentation of transition discussion, transfer to adult neurology, and retention in adult neurology. Candidate predictors included in all models were baseline age, sex, white non-Hispanic versus other race/ethnicity, documented ID, documented use of tracheostomy or feeding tube, documented DRE, use of ketogenic diet, use of a vagus nerve stimulator, history of resective/ablative surgery for epilepsy treatment, distance of zip code from the medical center in miles, zip code median household income (categorized as up to $55,000, $55,000 to $89,744, and over $88,744), and zip code rurality. For transfer to and retention in adult neurology, presence of documentation of transition discussion was also included. We dichotomized race/ethnicity due to small numbers of individuals identifying as races or ethnicities other than white non-Hispanic, reflecting the demographics of the institution’s region.^23^ We did not include use of responsive neurostimulation in the model, as none of the patients in this sample used responsive neurostimulation. We determined the threshold of statistical significance to be an alpha level of 0.05.

## Results

### Sample characteristics

We evaluated 274 individuals with epilepsy aged at least 15 years seen by outpatient Child Neurology at our institution in 2019. The median age was 18 years (interquartile range, 16-19; range, 15-28). One hundred and thirty-six individuals (50%) were female, 237 (84%) were white, and four (1.5%) were Hispanic. Sixty-six (24%) were identified as having DRE, and 69 (25%) had a documented diagnosis of ID. The majority of patients (n = 253, 92%) did not require advanced therapeutics such as ketogenic diet, vagus nerve stimulator implantation, responsive neurostimulator implantation, or resective/ablative surgery as part of their epilepsy care. Similarly, most patients (n = 252, 92%) did not use technologies such as tracheostomy or feeding tube. We display the detailed sample characteristics in Table 1.

### Transition outcomes

Fourteen patients (5%) died during pediatric care, and 28 patients (10%) were discharged from epilepsy care due to resolution of their epilepsy (i.e., due to resolution of an age-limited syndrome such as childhood absence epilepsy or self-limited epilepsy with centrotemporal spikes). Of the remaining 232 patients, 115 (50%) transitioned to adult epilepsy care by 2022, whereas 93 (40%) were lost to follow-up and 24 (10%) had ongoing pediatric epilepsy care by the end of 2022.

Whether or not they completed the transition, 57 patients (25%) had documentation of discussion regarding transition from pediatric to adult epilepsy care. No patients (0%) had a formal documented transition summary in their chart.

Ninety-five of 115 (83%) individuals who transitioned to adult epilepsy care had adult care notes available for review. Of these 95 eligible individuals, 77 (81%) were retained in adult care, defined as at least one visit per year since transfer to adult epilepsy care.

### Characteristics of individuals who transitioned to adult epilepsy care

Of 232 eligible individuals, 115 (50%) (who were neither deceased nor discharged from epilepsy care) ultimately completed the transfer to adult care. The median age at the first appointment under adult care was 20 years (interquartile range, 19-22; range, 15-31). The number of days between the patient’s last pediatric visit and first adult visit was 375 days (interquartile range, 196-616; range, 10-1288). Seven patients (6% overall, 12% of all patients who identified as female) transitioned to adult care specifically due to becoming pregnant.

### Predictors of documentation of discussion of transfer to adult epilepsy care

Statistically significant predictors of documentation of transition discussion taking place between patient and provider included older age (adjusted odds ratio [aOR], 1.44; 95% confidence interval [CI], 1.22-1.70) and absence of documented ID (aOR, 4.17; 95% CI, 1.51-11.11). Race/ethnicity, distance to medical center, median zip code household income, zip code rurality, presence of DRE, and presence of tracheostomy and/or feeding tube in pediatric care were not statistically significant predictors (Table 2).

### Predictors of completed transition to adult epilepsy care versus continued pediatric care or loss to follow-up

Statistically significant predictors of completed transfer from pediatric to adult epilepsy care included older age (aOR, 1.19; 95% CI, 1.02-1.39), presence of documentation of transition discussion (aOR, 3.25; 95% CI, 1.48-7.13), and presence of DRE (aOR, 2.41; 95% CI, 1.10-5.28). Gender, race/ethnicity, distance to medical center, median zip code household income, zip code rurality, documented ID in pediatric care, and presence of tracheostomy and/or feeding tube in pediatric care were not statistically significant predictors (Table 3).

### Predictors of retention in adult care

We identified no significant predictors of retention in adult care including age, gender, race/ethnicity, presence of documentation of transition discussion, distance to medical center, median zip code household income, zip code rurality, presence of DRE, and documented ID in pediatric care (Table 4).

## Discussion

Our study found that about half of the eligible patients with epilepsy who were at least 15 years old in 2019 had definitive evidence of transfer to adult care by 2022, with a median age of 20 years at the time of transfer and a median time of 375 days between the final pediatric and the first adult neurology appointment, with over 80% of individuals retaining in adult care. About 40% of our sample was lost to follow-up. About 25% of the sample had documented transition discussions. Significant predictors of documented transition discussion included older age and absence of documented ID. Significant predictors of completed transition included older age, presence of DRE, and presence of documented transition discussion. We identified no statistically significant predictors of retention in adult care. Twelve percent of female individuals who transferred did so due to pregnancy. Rates for documented transition discussion and completed transition fell well below our hypothesized maxima of 50% and 80%, although retention exceeded our hypothesized maximum of 80%. These findings describe baseline outcomes in a single institution that, at the time, did not have any formal or coordinated transition of care process for patients with epilepsy. These findings generally demonstrate that, in the absence of formal health system initiatives, existing clinical guidelines for ideal transition of care may not be met in practice.

There are several areas where our institution, in the absence of a coordinated transition program, fell short of guideline-concordant care based on our results. One challenge is that a large number of individuals (approximately 40% of the sample) were lost to follow-up. Practice-level tracking and monitoring of youth, as recommended by Got Transition, would likely reduce this potentially adverse outcome.^1^ Although many of these youth likely transitioned to adult care on their own, or perhaps sought pediatric neurology care at other institutions, there was no documented care coordination or sharing of information, including medical records, to facilitate care transfers, potentially resulting in fragmented care.

Even for individuals with a documented transfer of care, there was no formal transmission of a documented transfer packet for any individual, as recommended by both Got Transition and the consensus statement about the neurologist’s role in the transition process, demonstrating poor care coordination.^1,2^ The median time between the last pediatric and the first adult appointment was 375 days—more than twice the three- to six-month window recommended by Got Transition.^1^ Perhaps unsurprisingly, documentation of a transition discussion did significantly predict documented completed transfer, attesting to the potential value of transition planning. However, transition discussions were rare, as has been found in previous literature on epilepsy transition of care.^24,25^

The lack of a coordinated transition of care program may have an adverse impact on particularly vulnerable populations, potentially including people with epilepsy with co-occurring ID. Individuals with ID were significantly less likely to have a documented discussion of transition of care in their health record, although not less likely to transfer care, attesting to a potential equity issue in the transition planning and coordination process. This inequity is all the more troubling given that individuals with ID have arguably more complex transition of care needs requiring more deliberate planning than for people without ID. For instance, the neurology consensus statement emphasizes the need for assessment of legal competency in advance of legal adulthood.^2^ Our findings echo prior qualitative research that child neurologists may delay transitions of care for patients with ID partly due to concerns that adult neurology practices may have fewer accommodations for patients with disabilities.^15,17,18,26,27^ This literature suggests that improving care for patients with ID will require improvements in the transition process in adult neurology alongside improved transition preparation in pediatric neurology.^15,17,18,26,27^

Lack of coordinated transition of care programs may also exacerbate the risks faced by youth of child-bearing potential with epilepsy. Twelve percent of female individuals with epilepsy in the sample transferred abruptly to adult care due to pregnancy, attesting to the importance of child neurologists delivering age-appropriate anticipatory guidance about puberty, sexuality, and reproductive health, as recommended in the neurologist consensus statement.^26^ Prior literature has attested to child neurologist overall omission of anticipatory guidance about reproductive health and epilepsy, or performance of such counseling in a low-quality manner.^28–34^

These study findings altogether suggest the need for coordinated processes for transition of care within institutions or practices, rather than leaving the transition process *ad hoc* entirely to the discretion of individual clinicians. Models of formal transition of care processes in epilepsy implemented in other institutions have included a “multidisciplinary day hospital” where the patient undergoes multiple assessments and educational activities, in conjunction with representatives from both pediatric and adult neurology.^5^ Another model is a “joint clinic” involving longitudinal combined visits with both pediatric and adult neurology.^7^ An additional model is integration of multiple qualitative improvement strategies, such as self-management education for youth by nurses, a Best Practice Advisory about transition in the EHR for clinicians, and creation of a transfer packet template for clinicians in the EHR.^6^ An additional possibility may be integration of a case manager for coordination of the transition process. Many of these models have been evaluated in terms of health care process outcomes, such as transfer rates to adult neurology within six months of the last pediatric appointment with favorable results.^5–7^ However, these programs have not been evaluated in terms of health outcomes such as seizure burden.

Given our study findings, our institution decided to develop an integrated clinic combining pediatric and adult epileptology for patients with epilepsy to facilitate the transition process. A mixed methods evaluation of outcomes from this clinic is currently underway. More research is needed on understanding optimal transition of care programs in epilepsy and neurology, not only on health process outcomes but also on clinical outcomes such as seizure burden, and implementation outcomes such as program sustainability, which may be challenging based on prior literature.^35^

Our study findings, in conjunction with findings from two prior institution-wide needs assessments in our pediatric health system, also attest to the potential need for institution-wide transition initiatives.^10,11^ In a prior qualitative study of transition of care within our institution, challenges identified by division leaders during interviews included the lack of a coordinated institutional approach to health care transition, as well as heterogeneity within and between transition programs.^10^ These prior studies suggest a widespread institutional culture of poor health care transition practices, as only 20% of respondents to a separate survey at our institution reported satisfaction with their team’s transition-related practices.^11^ Furthermore, in this survey, 45% of providers across specialties self-reported discussing health care transition at least annually with adolescent and young adult patients, compared with 25% of youth with epilepsy in this study who had a documented transition discussion in their EHR, overall attesting to the suboptimal frequency of transition discussion, with the discrepancies in these numbers likely attributable to differences in data collection methods.^11^ These findings together indicate that problems in health care transition are not unique to epilepsy or neurology care; institution-wide strategies, in addition to disease-specific or specialty-specific strategies, may be warranted.

This study has several limitations. One is that documentation of a transition discussion does not prove that such a discussion occurred and provides no information about the quality of such a discussion. Absence of such documentation also does not exclude that such a discussion did not occur. However, clinical documentation, nevertheless, reveals the clinician’s priorities and thought processes. It would therefore be reasonable to infer that clinicians who document transition discussions emphasize transition of care in their health care encounters more than those who do not, although these findings must be interpreted with caution. An additional limitation is that this study occurred in a single center and therefore does not reflect practices or outcomes at other centers. Nevertheless, our study findings may serve as an illustrative case study for other similar tertiary care/quaternary care institutions regarding the potential state of transition of epilepsy care in the absence of formal transition of care programs. Our study relies on retrospective analysis of EHR data, with data collection limited to variables available in the EHR. We had access to EHR data at the two major health systems that dominate adult neurological care in our region; however, unaffiliated private adult neurology practices exist that are likely few in number and see a relatively large volume of patients. We do not have access to outcomes for patients seen in these practices. In addition, future prospective studies of transition of care patterns are warranted that can examine additional variables that are not routinely collected in clinical care, such as patient and family quality-of-life assessment and detailed seizure monitoring. We were also not able, as a part of this study, to request how often patients, families, or adult practices requested patients’ medical records, which may also be a direction for future inquiry.

Additionally, due to provision of team-based care in our pediatric health system, we were unable to assess whether duration of follow-up with a single pediatric provider or type of provider (i.e., attending physicians, residents/fellows, and advanced practice providers) affected transition and transfer outcomes because of limited continuity with specific individual providers or types of providers. Finally, our study time period overlapped with the coronavirus disease 2019 pandemic and it is unclear how the pandemic may have influenced our findings. However, pediatric and adult neurological providers in our area pivoted quickly to telemedicine during the pandemic for patients with epilepsy, which to some extent preserved accessibility of appointments for routine follow-up care, including transfer.

In conclusion, we found that, in our institution in the absence of a formal transition of care program for youth with epilepsy, care for youth with epilepsy did not meet either existing general medical or neurology-specific transition of care best practice guidelines. Our findings attest to the need for a formal transition of care program to oversee and implement guideline-concordant care. However, more research is needed on different types of formal transition of care programs to determine the optimal models of care and implementation strategies to improve the transition and transfer process for patients with epilepsy.

## Figures and Tables

**TABLE 1.** Sample Characteristics

Variable (n = 274)	n (%) or Median (IQR)
Sex	
Male/assigned male at birth	138 (50%)
Female/assigned female at birth	136 (50%)
Age at 2019 outpatient visit	18 (16, 19), range 15-28
Race	
White or Caucasian	237 (84%)
Black or African American	29 (11%)
Multiracial	4 (1%)
Asian	2 (0.5%)
Unspecified	2 (0.5%)
Ethnicity	258 (94%)
Not Hispanic	4 (1.5%)
Hispanic	12 (4.5%)
Unspecified	
Zip code distance from medical center (miles)	27 (13, 61), range 0.8-963
Zip code median household income (US dollars)	
Up to $55,000	72 (26%)
$55,0001-$89,744	167 (61%)
Over $89,744	35 (13%)
Zip code rurality	
Not rural	199 (72.5%)
Rural	73 (27%)
Unspecified	2 (0.5%)
Drug-resistant epilepsy by ILAE criteria	
Drug-resistant epilepsy	66 (24%)
Epilepsy not drug resistant	208 (76%)
Advanced therapeutics	
Using ketogenic diet	3 (1%)
Vagus nerve stimulator implanted	13 (5%)
Responsive neurostimulation implanted	0 (0%)
Resective or ablative surgery	5 (2%)
No advanced therapeutics	253 (92%)
Intellectual disability	
Documented intellectual disability	69 (25%)
No documented intellectual disability	205 (76%)
Technology dependence	
Tracheostomy	5 (2%)
Feeding tube	15 (5%)
Cerebrospinal fluid shunt	11 (4%)
No technology dependence	252 (92%)
Outcome	
Lost to follow-up	93 (34%)
Ongoing pediatric care for epilepsy	24 (9%)
Discharged from care for epilepsy	28 (10%)
Deceased during pediatric care	14 (5%)
Transitioned to adult epilepsy care	115 (42%)
Documented transition summary	
Yes	0 (0%)
No	274 (100%)
Documented discussion of transfer to adult neurology if not deceased or discharged from care (n = 232)	
Yes	57 (25%)
No	169 (75%)

Abbreviation:

ILAE = International League Against Epilepsy

IQR = Interquartile range

**TABLE 2.** Predictors of Documentation of Discussion of Transfer to Adult Epilepsy Care

Variable	Adjusted Odds Ratio	95% Confidence Interval
Age*	1.44*	1.22, 1.70*
Gender		
Female	1.11	0.57, 2.14
Male	Reference	
Race		
Black, Asian, multiracial or Hispanic	0.87	0.30, 2.54
White non-Hispanic	Reference	
Distance to medical center	1.00	0.99, 1.01
Median zip code household income		
$89,745 and above	1.26	0.78, 4.08
$55,001-$89,744	1.78	0.36, 4.48
Up to $55,000	Reference	
Zip code rurality		
Rural	0.90	0.38, 2.10
Not rural	Reference	
Drug-resistant epilepsy by ILAE criteria	0.82	0.35, 1.91
Drug resistant	Reference	
Not drug resistant		
Documented intellectual disability in pediatric care*		
Intellectual disability	0.24*	0.09, 0.66*
No intellectual disability	Reference	
Presence of tracheostomy and/or feeding tube in pediatric care		
Tracheostomy or feeding tube	2.31	0.39, 13.70
Neither present	Reference	

Abbreviation:

ILAE = International League Against Epilepsy

* Denotes statistical significance at *P* < 0.05.

**TABLE 3.** Predictors of Completed Transition to Adult Epilepsy Care Versus Continued Pediatric Care or Loss to Follow-Up

Variable	Adjusted Odds Ratio	95% Confidence Interval
Age*	1.19	1.02, 1.39
Documentation of discussion of transfer to adult epilepsy care*		
Yes	3.25*	1.48, 7.13*
No	Reference	
Gender		
Female	1.15	0.64, 2.10
Male	Reference	
Race		
Black, Asian, multiracial, or Hispanic	1.26	0.51, 3.10
White non-Hispanic	Reference	
Distance to medical center	1.00	0.99, 1.01
Median zip code household income		
$89,745 and above	2.01	0.67, 6.01
$55,001-$89,744	0.99	0.49, 2.02
Up to $55,000	Reference	
Zip code rurality		
Rural	1.45	0.65, 3.23
Not rural	Reference	
Drug-resistant epilepsy by ILAE criteria*		
Drug resistant	2.41*	1.10, 5.28*
Not drug resistant	Reference	
Documented intellectual disability in pediatric care		
Intellectual disability	0.59	0.27, 1.31
No intellectual disability	Reference	
Presence of tracheostomy and/or feeding tube in pediatric care		
Tracheostomy or feeding tube	2.58	0.39, 17.27
Neither present	Reference	

Abbreviation:

ILAE = International League Against Epilepsy

* Denotes statistical significance at *P* < 0.05.

**TABLE 4.** Predictors of Retention in Adult Care (Defined as At Least One Adult Visit Per Year Post-Transfer)

Variable	Adjusted Odds Ratio	95% Confidence Interval
Age	0.85	0.61, 1.19
Documentation of discussion of transfer to adult epilepsy care		
Yes		
No	0.62	0.19, 1.98
Gender		
Female	0.88	0.25, 3.02
Male	Reference	
Race		
Black, Asian, multiracial, or Hispanic	0.53	0.10, 2.76
White non-Hispanic	Reference	
Distance to medical center	0.98	0.96, 1.00
Median zip code household income		
$89,745 and above	0.39	0.04, 3.51
$55,001-$89,744	0.40	0.08, 2.11
Up to $55,000	Reference	
Zip code rurality		
Rural	2.23	0.46, 10.75
Not rural	Reference	
Drug-resistant epilepsy during pediatric care by ILAE criteria		
Drug resistant	1.09	0.26, 4.64
Not drug resistant	Reference	
Documented intellectual disability in pediatric care		
Intellectual disability	7.26	0.71, 73.72
No intellectual disability	Reference	

Abbreviation:

ILAE = International League Against Epilepsy

Statistical significance at *P* < 0.05.
